# Hospital Meetings

**Published:** 1903-05-02

**Authors:** 


### HOSPITAL MEETINGS.

### METROPOLITAN HOSPITAL.

THE annual meeting of the governors of this hospital was held on April 27th. The chair was taken by Mr. C. J. Thomas, who, in his opening remarks, said that the report for the last year might be considered a record one, in view of the great advance that it chronicled in the amount of work done. The income, if not quite adequate for their requirements, was nearly so. The chief features of the past year were the increase in the work done and the arrangements that had been made in connection with the provident department. 1,285 in-patients had been treated in 1902, as against 1,131 in 1901. They had been able to assist a greater number of patients on account of the larger amount of beds available. The total of out-patients had also advanced from 39,431 in 1901, to 40,592 in the last year. With so large a number of attendances it was almost impossible to check them and to prevent people coming to the hospital who were not in every way deserving of assistance; it was in the endeavour to prevent any such abuse that they had developed the Provident Department and erected the neat building bearing that name on their own ground. This had been done only after long and careful consideration and at a cost of £1,191, which included building, furnishing, and equipment. The Chairman then acknowledged the great obligation the hospital was under to Mr. H. Lovegrove, who had designed and superintended the work and whose services were entirely gratuitous. To the careful attention he had bestowed upon the matter they owed in a great measure the success of the undertaking. An almoner had been appointed during the past year to inquire into the circumstances of the patients, and his services were of great advantage to the hospital in preventing people taking advantage of its benefits who were not entitled to them. The expenses during the year had been heavy, in fact the outlay had reached a higher figure than ever before, but this was due to the increased amount of work done. The committee exercised a strict supervision over the expenditure and endeavoured to be as economical as possible. Donations and legacies had decreased slightly.

It is noticeable in the report, although the fact was not referred to at the meeting, that the hospital had received a munificent grant of £2,500 from King Edward's Hospital Fund in addition to a subscription of £500, while increased grants were also made by both the Hospital Sunday and Hospital Saturday Funds. The committee were of opinion that to have obtained increased grants from each of these funds, which are known to be so carefully administered, was a proof that the "Metropolitan" had done good work in the past year, work which had met with the approval of those who best know how hospitals should be conducted.

